# Rapidly Escalating Hepcidin and Associated Serum Iron Starvation Are Features of the Acute Response to Typhoid Infection in Humans

**DOI:** 10.1371/journal.pntd.0004029

**Published:** 2015-09-22

**Authors:** Thomas C. Darton, Christoph J. Blohmke, Eleni Giannoulatou, Claire S. Waddington, Claire Jones, Pamela Sturges, Craig Webster, Hal Drakesmith, Andrew J. Pollard, Andrew E. Armitage

**Affiliations:** 1 Oxford Vaccine Group, Centre for Clinical Vaccinology and Tropical Medicine, Department of Paediatrics, University of Oxford, and National Institute for Health Research Oxford Biomedical Research Centre, Oxford, United Kingdom; 2 Victor Chang Cardiac Research Institute, Darlinghurst, New South Wales, Australia; 3 Department of Biochemistry, Birmingham Heartlands Hospital, Heart of England NHS Foundation Trust, Birmingham, United Kingdom; 4 BRC Blood Theme, NIHR Oxford Biomedical Research Centre, Oxford, United Kingdom; 5 MRC Human Immunology Unit, MRC Weatherall Institute of Molecular Medicine, University of Oxford, John Radcliffe Hospital, Oxford, United Kingdom; Oxford University Clinical Research Unit, VIETNAM

## Abstract

**Background:**

Iron is a key pathogenic determinant of many infectious diseases. Hepcidin, the hormone responsible for governing systemic iron homeostasis, is widely hypothesized to represent a key component of nutritional immunity through regulating the accessibility of iron to invading microorganisms during infection. However, the deployment of hepcidin in human bacterial infections remains poorly characterized. Typhoid fever is a globally significant, human-restricted bacterial infection, but understanding of its pathogenesis, especially during the critical early phases, likewise is poorly understood. Here, we investigate alterations in hepcidin and iron/inflammatory indices following experimental human typhoid challenge.

**Methodology/Principal Findings:**

Fifty study participants were challenged with *Salmonella enterica* serovar Typhi and monitored for evidence of typhoid fever. Serum hepcidin, ferritin, serum iron parameters, C-reactive protein (CRP), and plasma IL-6 and TNF-alpha concentrations were measured during the 14 days following challenge. We found that hepcidin concentrations were markedly higher during acute typhoid infection than at baseline. Hepcidin elevations mirrored the kinetics of fever, and were accompanied by profound hypoferremia, increased CRP and ferritin, despite only modest elevations in IL-6 and TNF-alpha in some individuals. During inflammation, the extent of hepcidin upregulation associated with the degree of hypoferremia.

**Conclusions/Significance:**

We demonstrate that strong hepcidin upregulation and hypoferremia, coincident with fever and systemic inflammation, are hallmarks of the early innate response to acute typhoid infection. We hypothesize that hepcidin-mediated iron redistribution into macrophages may contribute to *S*. Typhi pathogenesis by increasing iron availability for macrophage-tropic bacteria, and that targeting macrophage iron retention may represent a strategy for limiting infections with macrophage-tropic pathogens such as *S*. Typhi.

## Author Summary

An adequate supply of iron is essential for both human hosts and their infecting pathogens. Hepcidin is the human hormone that controls the quantity and distribution of iron throughout the body. During infections, hepcidin activity may redistribute iron away from serum and into macrophages, potentially affecting pathogen replication, depending on the niche of the invading microbe. However, the involvement of hepcidin in human bacterial infections remains poorly investigated. Similarly, the pathogenesis of typhoid fever, caused by infection with *Salmonella* Typhi is also poorly understood. We therefore investigated the behaviour of hepcidin and other iron/inflammation-related parameters during the course of typhoid fever in human volunteers challenged experimentally with *Salmonella* Typhi. Hepcidin concentrations rose rapidly during acute typhoid infection, in parallel with fever. Hepcidin induction was accompanied by a rapid decline in serum iron concentrations, likely reflecting iron sequestration in macrophages (a preferred replication site of *Salmonella* Typhi). The extent of hepcidin upregulation associated with the extent of serum iron starvation. We hypothesize that hepcidin activity during acute typhoid infection in humans may elevate iron levels in the niche used by the pathogen for replication. Targeting macrophage iron retention should be evaluated as a potential strategy for limiting typhoid fever.

## Introduction

Typhoid fever is a common infection that follows oral ingestion and invasion of the Gram-negative bacterium *Salmonella enterica* serovar Typhi (*S*. Typhi). An estimated 26.9 million cases occurred globally in 2010, disproportionately affecting children in resource-limited areas of sub-Saharan Africa and southeastern Asia [1,2].

*S*. Typhi is a human-restricted pathogen. Unlike non-typhoidal *Salmonella* infection, which is characterized by rapid-onset gastrointestinal inflammation and diarrheal illness in immunocompetent adults, *S*. Typhi causes a systemic infection. After ingestion, bacteria disseminate through the reticuloendothelial system, where they are thought to incubate for 7–14 days. Clinical illness then develops, characterized by fever and non-specific symptoms including headache, nausea and abdominal pain, and accompanied by bacteremia [3]. However, detailed understanding of typhoid pathogenesis remains limited, in part since convincing small-animal infection models are lacking [4]. An experimental human *S*. Typhi challenge model was recently reestablished, presenting a unique opportunity to investigate typhoid pathogenesis in a controlled setting in the natural host [5–7].

Conflict exists between hosts and invading pathogens over the control of the critical micronutrient, iron (reviewed in [8,9]). To limit free iron availability, mammalian hosts sequester iron using high-affinity iron-binding proteins including transferrin, lactoferrin, haptoglobin, hemopexin and the iron storage protein ferritin. To counteract this, many bacteria express higher affinity siderophores (e.g. enterobactin) that appropriate iron from host iron binding proteins; host-produced siderophore-binding proteins such as lipocalin-2 in turn counter these. A further host response to infection involves the rapid induction of hypoferremia, where iron becomes sequestered in reticuloendothelial macrophages and therefore excluded from serum [8,10]. This state may be disadvantageous to extracellular pathogens [11] but potentially could be exploited by intracellular, macrophage-tropic bacteria including *S*. Typhi [12]. Together, the host mechanisms aimed at sequestering iron from invading microorganisms are considered to contribute to innate protection against infection, often termed “nutritional immunity” [8].

In recent years, many genes involved in mammalian iron homeostasis have been discovered [13], meaning that the molecular basis of iron perturbations during infections can be investigated in a new light. Amongst these, hepcidin stands out as the central regulator of systemic iron balance [14]. Hepcidin dictates dietary iron uptake and recycling of red cell iron by binding and causing degradation of the sole known iron exporter ferroportin, which is expressed on duodenal enterocytes and iron-recycling macrophages [15]. Consequently, high hepcidin levels effect iron exclusion from serum, through blocking dietary iron uptake and preventing macrophage iron release. Hepcidin is induced homeostatically in response to increased plasma and liver iron [16,17], but is also an acute phase protein upregulated by inflammatory cytokines, notably IL-6 [18–20]. Thus, elevated hepcidin concentrations during inflammation and infections contribute to hypoferremia [11,21] and, if chronic, to iron-restricted erythropoiesis and anemia [22].

Hepcidin regulation is less well studied in the context of human infection. Analyses to date indicate that hepcidin behavior differs between infections. For example, it is upregulated during uncomplicated malaria [23–26], and during acute, chronic and advanced HIV-1 infection [27,28]; however, it is suppressed during Hepatitis C Virus infection [29], and in severe malarial anemia, where bone-marrow derived signals indicating erythropoietic iron demand likely dominate, suppressing hepcidin production [26,30]. Importantly, hepcidin remains remarkably poorly studied in human bacterial infections. This is despite iron representing a battleground of host-bacterial conflict important enough to have shaped both primate and bacterial genomes alike [31]. Here, we investigate the dynamics of hepcidin in relation to iron and inflammatory indices during acute experimental *Salmonella* Typhi infection in humans.

## Methods

### Study participants

Human typhoid challenge was performed with healthy consenting adult volunteers (18–60 years) who had not previously received typhoid vaccination or resided in typhoid-endemic areas for >6 months [7]. Data from two sets of study participants are described: first, from the placebo arm of a vaccine/typhoid challenge study, where participants received an oral placebo vaccine (sodium bicarbonate solution and excipients) 28 days before oral challenge with *S*. Typhi (*n* = 30, Study A, Table 1 (baseline data from day of typhoid challenge shown)), and secondly, for more detailed longitudinal analysis, from a previously described cohort challenged orally with *S*. Typhi in a preliminary dose-escalation challenge model (*n* = 20, Study B, Table 1) [7].

**Table 1:** Baseline characteristics of study populations on the day of typhoid challenge.

Parameter	Study A (placebo arm of vaccine/challenge study)	Study B (challenge study)
Number of participants	30	20
Age, median years (IQR) ^b^	24.6 (21.7–39.4)	26.5 (23.4–38.4)
Male gender, *n* (%) ^b^	18 (60)	12 (60)
Weight, median kg (IQR) ^b^	75.9 (68.9–82) (n = 18)	79.4 (71.7–87.0) (n = 15)
Height, median m (IQR) ^b^	1.71 (1.67–1.82)	NA
Challenge dose, mean Log_10_ ^a^	4.27 (±0.07)	4.30 (±0.06)
Typhoid diagnosed, *n* (% of study) ^b^	20 (66.6)	13 (65)
Days to typhoid diagnosis, median number (range) ^b^	7 (5–11)	8 (5–10)
Hemoglobin, ^b^	14.1 (±1.6)	14.0 (±1.3)
Mean Corpuscular Volume (MCV), ^b^	91.06 (± 4.61)	90.61 (±4.79)
Hematocrit, ^b^	0.428 (±0.043)	0.428 (±0.037)
Platelets, ^b^	248.5 (±64.9)	226.5 (± 38.9)
White cell count, ^b^	6.2 (±1.8)	6.5 (±2.3)
Neutrophils, ^b^	3.40 (±1.38)	3.59 (± 2.16)
Lymphocytes, ^b^	2.01 (±0.65)	2.09 (± 0.59)
Eosinophils, ^b^	0.21 (±0.11)	0.29 (± 0.15)
Serum Iron, μmol/L, ^b^	12.3 (±5.6)	14.9 (±5.2)
Total Iron Binding Capacity (TIBC), μmol/L ^a^	56.3 (±7.2)	51.8 (±8.2)
Transferrin saturation (Tsat), %, ^a^	22.3 (±10.6)	29.0 (±9.4)
Log_10_ ferritin, μg/L, ^b^	1.61 (0.38)	1.71 (0.37)
Log_10_ hepcidin, ng/mL, ^b^	1.01 (0.34)	1.1 (0.28)
Log_10_ CRP, mg/L ^b^	0.10 (±0.51)	-0.29 (±0.57)

^a^ p<0.05, t test

^b^ p>0.05

^c^ arithmetic mean (+/- standard deviation) reported unless stated otherwise.

### Typhoid challenge model

Full details of the challenge model used in both studies are described in Waddington *et al* [7]. Briefly, participants ingested a single freshly prepared dose of *S*. Typhi (Quailes strain, 10^4^ CFU) suspended in sodium bicarbonate solution. After challenge, study participants were reviewed daily for 14 days; blood samples were collected on alternate days, at typhoid diagnosis and intervals thereafter. In typhoid-infected and non-infected participants, the mean blood volumes collected during the 28 days following challenge were approximately 920mL and 600mL respectively. “Typhoid diagnosis” was defined *a priori* by clinical and/or microbiological endpoints: temperature ≥38°C sustained for ≥12 hours and/or blood culture evidence of *S*. Typhi bacteremia, respectively. Antibiotic treatment (ciprofloxacin, 500 mg twice daily, 14 days) was initiated upon attainment of either diagnostic criterion and in all remaining participants at Day 14. Actual challenge doses were determined, and quantitative blood culture was performed at typhoid diagnosis, as previously described [7].

### Quantification of hepcidin and other analytes

Serum samples were filtered prior to analyses using Costar Spin-X low protein binding 0.22μm cellulose acetate membrane filters (Corning). Spin-filtering had no effect on hepcidin measurement (n = 4 samples, *p* = 0.36 (paired t-test)). Hepcidin was quantified by ELISA using the hepcidin-25 EIA kit (Bachem), with the manufacturer’s protocol modified to incorporate a 9-point, 2-fold serial dilution standard curve. Samples were diluted to 10% or 5% prior to analysis. The lower limit of detection (LLOD) was 0.08 ng/mL, calculated as described previously [27]. Samples returning a reading below LLOD were assigned the value (LLOD*dilution factor)/2.

Serum ferritin (Architect Ferritin Assay) was quantified using the Abbott Architect 2000R automated analyzer (Abbott Laboratories); C-reactive protein (CRP) (MULTIGENT CRP Vario Kit, with high sensitivity calibrators), serum iron and Unsaturated Iron Binding Capacity (UIBC, MULTIGENT Iron Kit) were quantified using the Abbott Architect c16000 automated analyzer (Abbott Laboratories). Transferrin saturation (Tsat) was calculated using the formula: Tsat = ((Serum Iron)/(Serum Iron + Unsaturated Iron Binding Capacity))*100. CRP concentrations above and below the assay limits of detection (160 and 0.1 mg/L) were assigned the values 160 mg/L and 0.05 mg/L respectively.

Plasma cytokine concentrations were measured in duplicate using a custom TNFα / IL-6 Luminex panel (MILLIPLEX MAP kit, Millipore) according to the manufacturer’s instructions. Readings with % Coefficient of Variance >30% were excluded; those falling below the LLOD were allocated the value 1.6 pg/mL (LLOD/2).

Hemoglobin, red blood cell counts, and mean corpuscular volume (MCV) were quantified by routine hematologic analysis.

### Statistical analysis

Statistical analyses were performed using Prism (version 6, GraphPad Software Inc.), SPSS (version 16.0, IBM SPSS), STATA/SE13.1 (Statacorp) and R statistical language [32]. All raw data can be found in the file S1 Dataset.

For indices that were not normally distributed (hepcidin, ferritin, and CRP in all cases; additionally serum iron and transferrin saturation when considering data other than baseline data), geometric means were compared, or data were log-transformed prior to analysis. Differences between indices pre-challenge and at typhoid diagnosis (Study A) were evaluated using paired t-tests. In correlation analysis, Pearson correlation coefficients were computed; in cases where study participants contributed more than a single observation, correlation analyses were adjusted accordingly by using regression with clustered errors (STATA/SE13.1), which adjusts the confidence intervals of the regression coefficients to account for intra-cluster correlation, as is likely when multiple observations from the same individuals are included. Statistical tests returning *p*<0.05 were considered significant.

Time-course analyses were performed using the packages *fields* [33], *nlme* [34] and *lme4* [35] within R statistical language [32] as follows. *(i) Normalization of time series*: Since the time between typhoid challenge and diagnosis (TD) varied between participants, the time variable “day relative to TD” was used, with TD = 0 being day of diagnosis. *(ii) Smoothing spline regression*: Mean analyte measurements across all subjects for each day relative to TD were calculated; samples from day of typhoid challenge (‘baseline’) and the final visit (Day 14 post-challenge) were grouped separately. A smoothing spline regression was applied with smoothness estimated from the data by generalised cross validation (GCV) [36]. 95% pointwise prediction intervals and conservative simultaneous Bonferroni bounds were calculated. (*iii) Assessment of the effect of time relative to TD on analyte concentrations*: linear mixed-effects models were fitted using a described model formulation [37] and computational framework [38]. The categorical variable “day relative to TD” was modeled by fixed effects; variability between individuals was captured using random effects. The null hypothesis that there is no significant difference in analyte levels over time after challenge was tested using the Wald test; specific pairwise comparisons between analyte concentrations at baseline (day of challenge) and later time-points were tested by t-tests, accounting for subject-specific variability. Tests returning p<0.05 were considered significant.

### Ethics

The National Research Ethics Service approved both studies (Oxfordshire Research Ethics Committee A, 10/H0604/53 and 11/SC/0302). They were performed in accordance with the principles of the ICH-Good Clinical Practice guidelines and amendments. All study participants provided written informed consent in accordance with the Declaration of Helsinki on at least one occasion, as previously described [7].

## Results

### Baseline characteristics of study participants

Characteristics of study participants from two experimental typhoid challenge studies are given in Table 1. The typhoid attack rates (percent typhoid-diagnosed participants by Day 14) in Study A and B were 67% (20/30) and 65% (13/20), while mean duration between challenge and typhoid diagnosis was 7.4 (95% Confidence Interval (CI): 6.6–8.3 days) and 7.7 days (6.7–8.7 days), respectively. There were no significant differences in the baseline characteristics of participants recruited to Study A or B, except that the challenge dose and transferrin saturations were marginally higher in Study B. Considering all participants from Studies A and B together, significant associations between log_10_-hepcidin and log_10_-ferritin levels (p<0.0001, r^2^ = 0.642; S1 Fig, panel A) and between hepcidin and both transferrin saturation (S1 Fig, panel B) and hemoglobin (S1 Fig, panel C) were found in baseline, pre-challenge samples. Male participants had significantly higher baseline hemoglobin, hepcidin and ferritin levels than females (S1 Fig, panels D-F). These observations are typical of healthy adult populations.

### Effect of baseline characteristics on typhoid susceptibility

In univariate analyses, there were no significant differences in age, sex, weight or challenge dose, or in baseline hematological or iron-related parameters between those subsequently diagnosed or not diagnosed with infection, even when participants from the two studies were pooled together to increase power (S1 Table). Amongst individuals diagnosed with typhoid, we found no association between the time to typhoid diagnosis and baseline iron status as indicated by ferritin (r^2^ = 0.014, *p* = 0.505) or hepcidin (r^2^ = 0.015, *p* = 0.497). Amongst individuals from the two studies who were diagnosed with typhoid, increasing challenge dose was significantly negatively associated with time-to-diagnosis (r^2^ = 0.174, *p* = 0.016) and positively associated with the number of bacteria quantified at diagnosis (r^2^ = 0.241, *p =* 0.007).

### Hepcidin is significantly elevated following typhoid diagnosis

**Fig 1:**
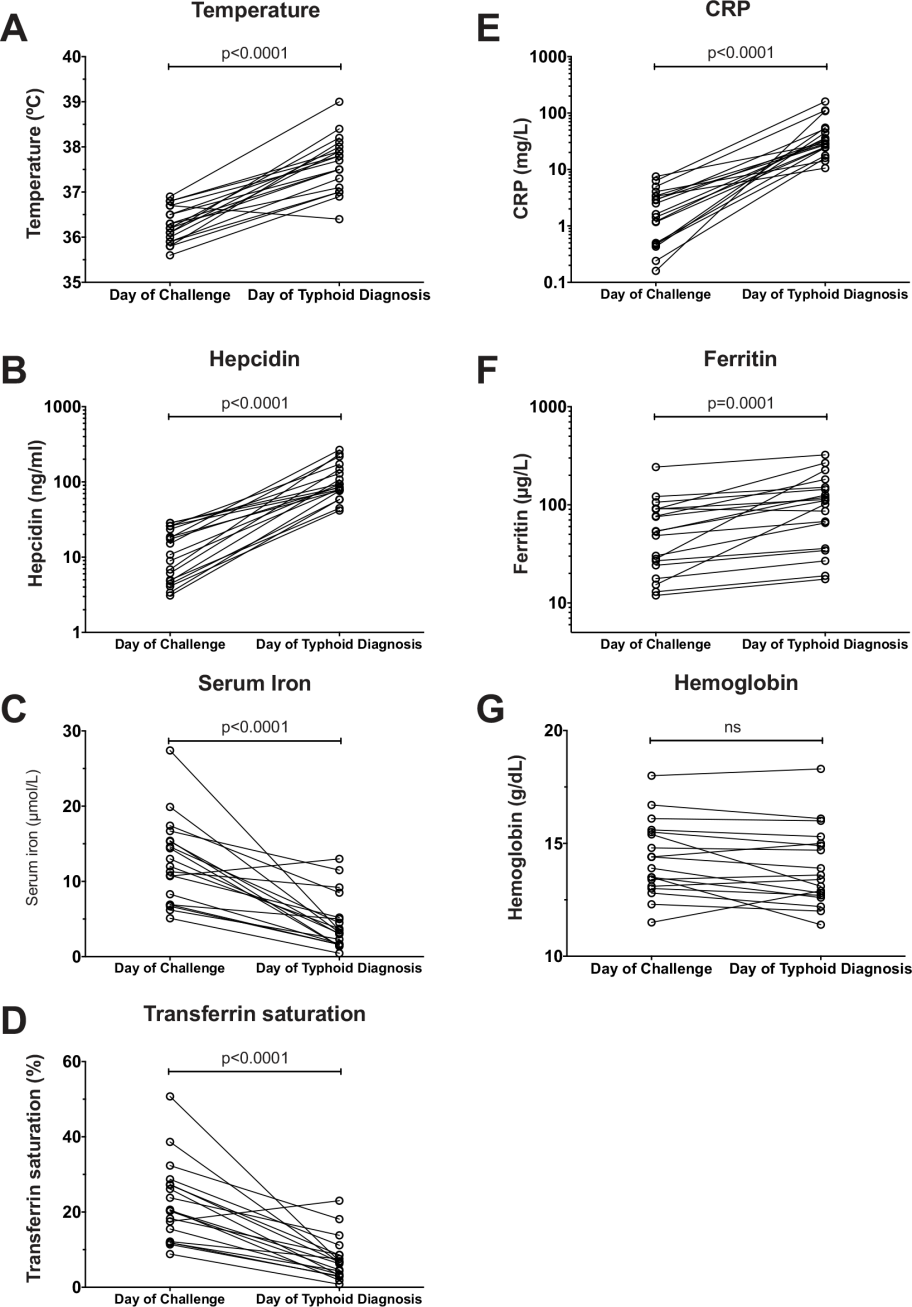
Changes in hepcidin, iron and inflammatory indices between baseline–the day of typhoid challenge–and the day of typhoid diagnosis. Serum samples were available from both day of typhoid challenge and day of typhoid diagnosis from 19 individuals from Study A (placebo arm of vaccine/typhoid challenge study). (A) temperature, (B) hepcidin, (C) serum iron, (D) transferrin saturation, (E) CRP, (F) ferritin and (G) hemoglobin were measured on the day of challenge and day of typhoid diagnosis. P-values represent results of paired t tests based on geometric means for hepcidin (baseline, 10.4 ng/mL [95% CI: 7.1–15.3]; diagnosis, 98.2 ng/mL [75.9–126.9]), ferritin (baseline, 46.3 μg/L [30.8–69.8]; diagnosis, 86.4μg/L [56.9–131.0]) and CRP (baseline: 1.46 mg/L [0.84–2.54]; diagnosis, 34.1 mg/L [24.2–48.2]), and arithmetic means for temperature (baseline, 36.3°C [36.1–36.5]; diagnosis, 37.6°C [37.3–37.9]), serum iron (baseline, 12.6 μmol/L [9.9–15.3]; diagnosis, 4.4 μmol/L [2.7–6.1]), transferrin saturation (baseline, 22.3% [17.2–27.4]; diagnosis, 7.4% [4.7–10.2]) and hemoglobin (baseline, 14.3 g/dL [13.6–15.1]; diagnosis 13.9 g/dL [13.1–14.8]).

To investigate the extent to which typhoid infection was associated with changes in hepcidin and other iron indices, we analyzed serum samples collected at baseline and on day of typhoid diagnosis in participants challenged in Study A (*n* = 19/20, 7 females and 12 males). Amongst these individuals, the mean time to typhoid diagnosis was 7.4 days (95% CI: 6.6–8.3 days); mean oral temperature was significantly higher at typhoid diagnosis than at baseline (diagnosis: 37.6°C [95% CI, 37.3–37.9°C]; baseline: 36.3°C [36.1–36.5°C]; Fig 1A).

Hepcidin concentrations at typhoid diagnosis were approximately 10-fold higher than at baseline (Fig 1B). This marked hepcidin response was accompanied by hypoferremia demonstrated by a significant decline in mean serum iron and transferrin saturation (Fig 1C and 1D). In contrast, there was a significant increase in the inflammatory markers, CRP and ferritin at diagnosis compared to baseline, although the relative change in ferritin concentration was less notable than that of hepcidin or CRP (Fig 1E and 1F). There were no significant differences in hemoglobin between measurements at baseline and at diagnosis (Fig 1G). Together, these data demonstrate that significant hepcidin upregulation and concurrent hypoferremia are features of the acute phase response to *S*. Typhi infection.

### Kinetics of hepcidin and iron perturbations in typhoid-challenged participants who did not develop infection

To assess the kinetics of alterations in hepcidin and other indices following *S*. Typhi challenge, we analyzed serial samples from Study B, firstly from the 7 participants who did not develop clinical disease following challenge, and secondly from the 13 individuals diagnosed with acute infection. For both groups, up to 7 time points from Day 0 (baseline, challenge day) onwards were analyzed (mean, 6.15 time points).

In those who did not develop typhoid infection, significant reductions in hepcidin, ferritin and hemoglobin concentrations, and in red blood cell counts, were observed during the 14-day study period (S2 Fig, panels A-D). There was also suggestion of decline in serum iron and transferrin saturation (S2 Fig, panels E/F). This likely relates to the repeated phlebotomy required by the study protocol, causing reduction of iron indices including hepcidin. CRP concentrations and oral temperatures remained low/normal throughout confirming the absence of a systemic inflammatory response in challenged but non-infected individuals (S2 Fig, panels G/H). Thus, the following time course data from typhoid-infected individuals must be interpreted in the light of these study protocol effects on hematological parameters.

### Kinetics of hepcidin, iron and inflammatory perturbations during acute typhoid fever

**Fig 2:**
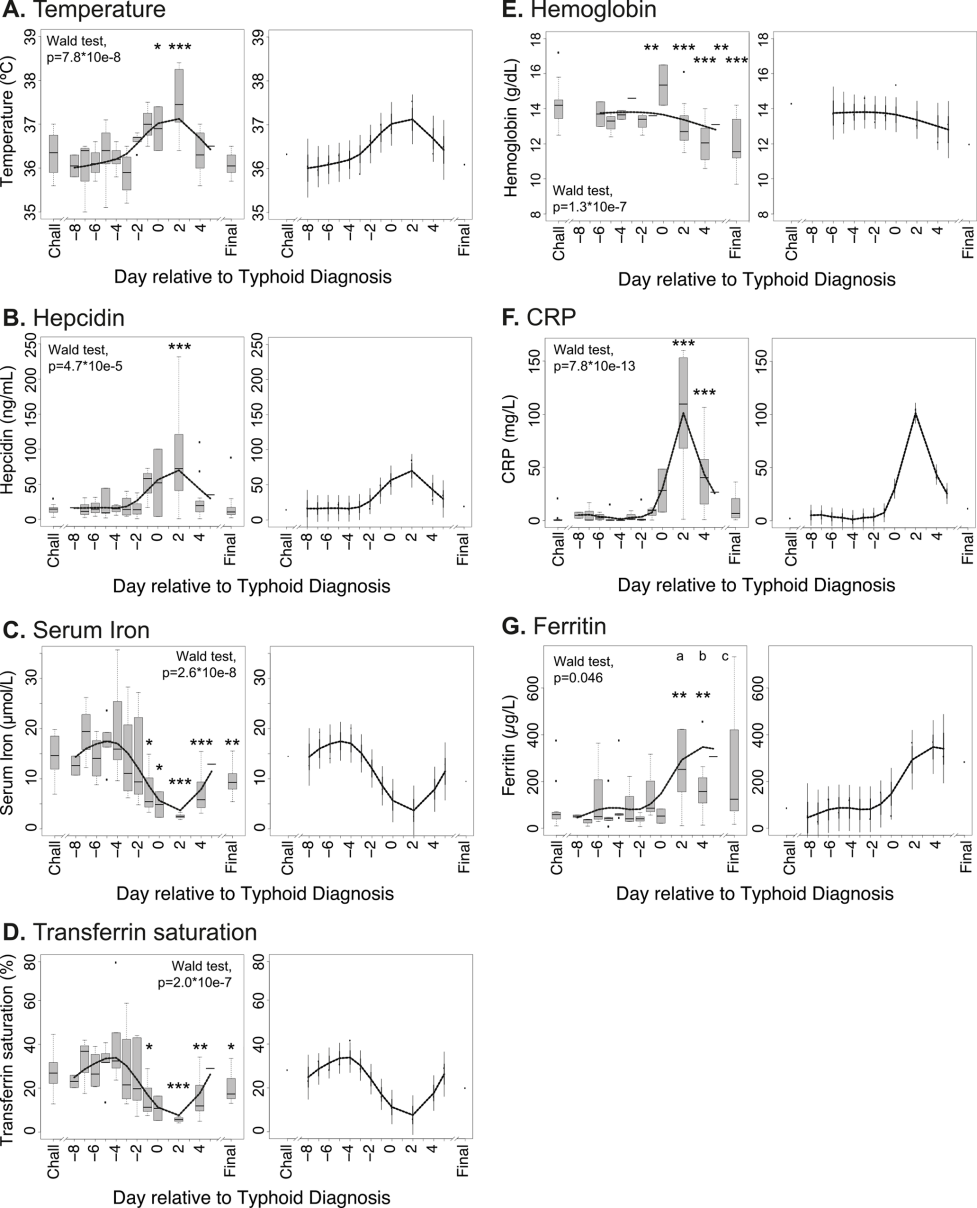
Kinetics of perturbations in hepcidin, iron and inflammatory parameters in individuals diagnosed with typhoid infection following experimental *Salmonella* Typhi challenge. (A) Temperatures, (B) serum hepcidin concentrations, (C) serum iron, (D) transferrin saturations, (E) hemoglobin, (F) CRP, and (G) ferritin concentrations were measured in 13 individuals from Study B who received typhoid diagnosis following challenge with *Salmonella* Typhi. Analyte values are plotted relative to the day of typhoid diagnosis, TD = day 0; since not all individuals were diagnosed on the same day post-challenge, baseline samples from the day of challenge (Chall) are considered together, as are data from the final day 14 visit (Final). (Left-hand panels) Data available from each individual for each day were plotted using box and whiskers, representing median values and interquartile ranges (IQR); whiskers represent the data point occurring furthest from the first or third quartile but still within 1.5*IQR of the quartile; outliers (further than 1.5*IQR from the quartile) are shown as isolated data points. Smoothed curves were also interpolated from the mean data for each day and overlaid on the plots. The Wald test was employed after fitting linear mixed effects models to test the null hypothesis that there is no difference between parameter values between days. Pairwise differences between baseline values on the day of typhoid challenge (Chall) and other days were examined by t-tests after accounting for subject-specific variability. Significant perturbations from baseline are indicated with asterisks (*p<0.05, **p<0.01, ***p<0.001). For ferritin, outliers at (a) 1014.07 μg/L and 1075.24 μg/L, (b) 2433.54 μg/L, and (c) 1008.87 μg/L are beyond the y-axis limits and not depicted on the figure, but are included in the analysis. (Right-hand panels) Smoothed interpolated curves as described above, but depicting 95% pointwise prediction intervals (thick error bar) and conservative simultaneous Bonferroni bounds (thin error bar) of the interpolated curves.

In participants who developed typhoid fever, increases in temperature were measured from 48 hours prior to diagnosis (Fig 2A). A concomitant rise in hepcidin concentration was observed, maximal 2 days after diagnosis; temperature and hepcidin levels normalized towards baseline levels over approximately 4 days following treatment initiation (Fig 2B). Similarly, significant declines in serum iron (commencing prior to diagnosis and reaching a mean nadir of 4.9 μmol/L two days post-diagnosis, down from 14.5 μmol/L at baseline, Fig 2C) and transferrin saturation (mean 6% at nadir two days post-diagnosis, down from 28% at baseline, Fig 2D) were observed; these indices, like hemoglobin (Fig 2E), were lower at the final time point (Day 14) than at baseline (Fig 2C and 2D and 2E), likely reflecting the effect of venesection described above (S2 Fig). However, the possibility of a hepcidin-mediated block in iron absorption during infection contributing to this observation should not be excluded.

A significant induction of the acute phase protein CRP was also observed, escalating marginally later than the initial perturbations to hepcidin and transferrin saturation, but similarly peaking 2 days after typhoid diagnosis (Fig 2F). The iron storage protein ferritin, also an acute phase protein, was induced later than hepcidin or CRP and took longer to resolve towards baseline levels (Fig 2G).

Together, these data indicate that the kinetics of hepcidin perturbations and the associated hypoferremia during acute *S*. Typhi infection mirror typhoid-associated fever and CRP induction.

### Contrary associations between hepcidin and iron parameters, according to inflammation status

**Fig 3:**
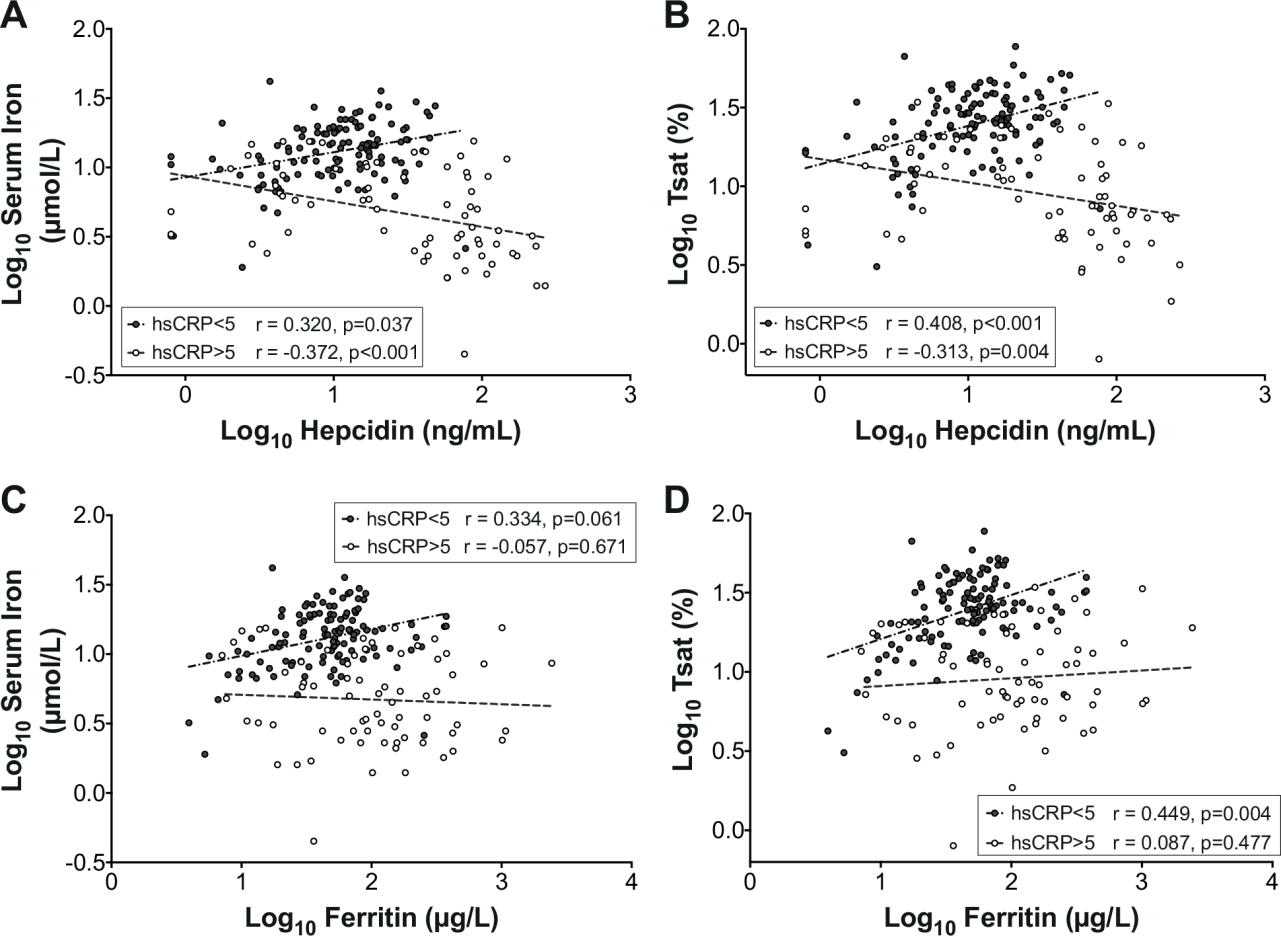
Opposite associations of hepcidin with serum iron and transferrin saturation in the presence and absence of inflammation. Associations between (A) hepcidin and serum iron, (B) hepcidin and transferrin saturation (Tsat), (C) ferritin and serum iron, and (D) ferritin and Tsat, when acute inflammation (defined as CRP>5 mg/L, open circles) or when no inflammation (CRP<5 mg/L, closed circles) was evident. The analysis considered all data obtained between challenge day and Day 14 post-challenge for each study participant, whether diagnosed with typhoid or not. To normalize data, each parameter was log-transformed prior to analysis. The analysis accounts for individuals contributing more than single data points by using regression with clustered errors (Serum Iron analyses (A) and (C): CRP <5 mg/L: 118 observations, 47 clusters; CRP >5 mg/L: 69 observations, 36 clusters. Tsat analyses (B) and (D): CRP <5 mg/L: 117 observations, 46 clusters; CRP >5 mg/L: 69 observations, 36 clusters). Regression with clustered errors adjusts the confidence intervals of the regression coefficients to account for intra-cluster correlation, as is likely when multiple observations from the same individuals are included. Pearson correlation coefficients and p-values are stated.

We next investigated relationships between hepcidin concentration and serum iron status in those exhibiting typhoid-related inflammation and those who were not. In this analysis, we included all data from the study from both diagnosed and non-diagnosed individuals, using regression with clustered errors, thereby accounting for the inclusion of multiple observations derived from the same individuals. When there was evidence of acute inflammation (defined as CRP >5 mg/L), significant negative associations between hepcidin and both serum iron and transferrin saturation were observed (Fig 3A and 3B). In contrast, when acute inflammation was absent (CRP <5 mg/L), significant positive associations between hepcidin and serum iron parameters were found (Fig 3A and 3B). Thus, larger hepcidin responses predicted more profound hypoferremia in the context of inflammation, but the opposite in non-inflamed samples, when they presumably reflected iron status. The latter effect was also noted in baseline challenge day samples (S1 Fig). Unlike hepcidin, ferritin did not correlate with the extent of hypoferremia during inflammation, although it did associate positively with serum iron parameters in non-inflamed samples (Fig 3C and 3D). These data indicate non-equivalence of these two indices of iron status, as noted in previous work [39], and suggest hepcidin may be more closely linked to hypoferremia in the context of the acute inflammation observed during typhoid infection.

### Modest induction of inflammatory cytokines in individuals with typhoid infection

**Fig 4:**
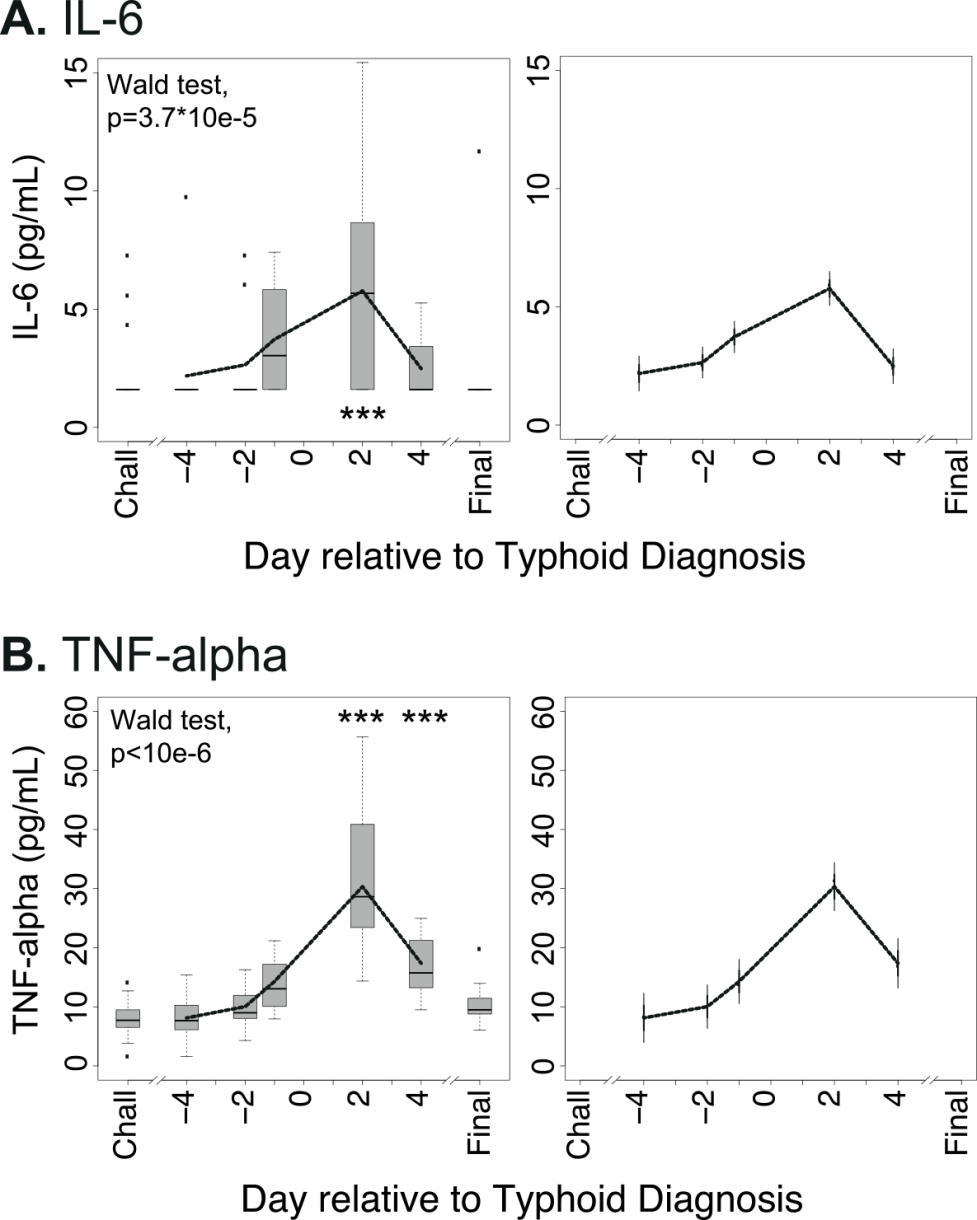
Kinetics of perturbations in IL-6 and TNF-alpha in individuals diagnosed with typhoid infection following experimental *Salmonella* Typhi challenge. (A) Plasma IL-6 and (B) TNF-alpha concentrations were measured in 13 individuals from Study B who received typhoid diagnosis following challenge with *Salmonella* Typhi. Analyte values are plotted relative to the day of typhoid diagnosis, TD = day 0; since not all individuals were diagnosed on the same day post-challenge, baseline samples from the day of challenge (Chall) are considered together, as are data from the final day 14 visit (Final). (Left-hand panels) Data available from each individual for each day were plotted using box and whiskers, representing median values and interquartile ranges (IQR); whiskers represent the data point occurring furthest from the first or third quartile but still within 1.5*IQR of the quartile; outliers (further than 1.5*IQR from the quartile) are shown as isolated data points. Smoothed curves were also interpolated from the mean data for each day and overlaid on the plots. The Wald test was employed after fitting linear mixed effects models to test the null hypothesis that there is no difference between parameter values between days. Pairwise differences between baseline values on the day of typhoid challenge (Chall) and other days were examined by t-tests after accounting for subject-specific variability. Significant perturbations from baseline are indicated with asterisks (***p<0.001). (Right-hand panels) Smoothed interpolated curves as described above, but depicting 95% pointwise prediction intervals (thick error bar) and conservative simultaneous Bonferroni bounds (thin error bar) of the interpolated curves.

Hepcidin upregulation during acute phase responses is typically associated with STAT3 activation following signaling by IL-6 and potentially other cytokines (e.g. IL-22) [18–20]. We therefore assessed IL-6 concentrations in plasma samples from the individuals from Study B who developed typhoid infection. We only observed a weak IL-6 response in a subset of individuals (Fig 4A, see S3 Fig for individual profiles); in the majority of individuals, IL-6 upregulation was not detected. Modest TNF-alpha responses were more consistent, with the highest levels recorded day 2 post-diagnosis in most individuals (Fig 4B, see S3 Fig for individual profiles). These data suggest the cytokine response during typhoid infection may have been blunted, as previously described [40], and that determinants other than serum IL-6 may be responsible for the hepcidin upregulation observed in this context.

## Discussion

*Salmonella* Typhi is a significant human pathogen, leading to a major global burden of disease particularly among children and younger adults in endemic settings [1,2]. Evolution from a common *Salmonella* ancestor is thought to have occurred ~50–100,000 years ago [41]. However, the basis for the evolution of its ability to evade host defenses and cause systemic infection remains poorly characterized. Understanding how *S*. Typhi interacts with the human host environment, including the macrophage niche, is crucial in deciphering its pathogenicity and for devising prevention or eradication strategies. The battle for iron is a key determinant of host-bacterial interactions [8,9,31]. Here, using an experimental human typhoid challenge model, we track for the first time in an invasive human bacterial infection the behavior of the iron regulatory hormone hepcidin and its relationship to perturbations in iron parameters, inflammatory markers, and fever: significant hepcidin upregulation, accompanied by a profound decline in serum iron was observed in participants diagnosed with typhoid infection.

Hepcidin has several characteristics reflecting a likely ancestry in immunity to infection. It is a liver-derived acute-phase peptide induced via the inflammatory JAK/STAT3 signaling pathway [18–20,42]. It structurally resembles antimicrobial beta-defensins and has modest antimicrobial activity itself [43,44]. Hepcidin’s involvement in human infection pathogenesis has been widely proposed, likely relating more to its ability to rapidly alter systemic partitioning of iron than its direct antimicrobial activity [12]. Despite this, its regulation and influence on the pathogenesis of human bacterial infection remains poorly investigated. In humans, significant hepcidin upregulation has been observed during sepsis [45,46], during tuberculosis (with and without HIV coinfection) [47,48], and to a less notable extent in children with concurrent *Helicobacter pylori* infection and iron deficiency anemia [49]. The longitudinal behavior of hepcidin has been assessed during experimental uncomplicated malaria (where modest increases in hepcidin and IL-6, associated with changes in systemic iron parameters, were observed) [23] and during the acute phases of HIV-1, Hepatitis B Virus and Hepatitis C Virus infections [27]. However, the nature of longitudinal perturbations in hepcidin during the acute phase of a bacterial infection in humans has never been investigated.

In study participants diagnosed with acute typhoid infection, we found a marked upregulation of hepcidin around the time of diagnosis, coincident with appearance of fever. Hepcidin concentrations remained high for at least 48-hours during acute infection irrespective of prompt antibiotic therapy, and resolved to normal levels from 4 days after diagnosis. We predict that hepcidin would remain high for considerably longer if the infection were left untreated. Significant elevations in the acute phase proteins, CRP and ferritin, and striking declines in serum iron and transferrin saturation (from 28% at baseline to 6% at nadir 2 days post-diagnosis) were also evident. The data suggested hepcidin activity from 1–2 days prior to typhoid diagnosis, and are consistent with the previous description of hypoferremia in the Maryland typhoid challenge in the 1970s [50]. Furthermore, our data indicated that, when acute inflammation was present, the extent of hepcidin upregulation significantly predicted the degree of hypoferremia; in contrast, in normal non-inflamed conditions, hepcidin positively associated with serum iron parameters. Given these data, hepcidin and the associated hypoferremia should be considered for investigation as potential biomarkers of acute infection.

Hepcidin upregulation in the context of inflammation/infection is typically linked to signaling via the IL-6/STAT3 pathway [19,42]. However, we only detected a modest elevation in plasma IL-6 around typhoid diagnosis, with several participants maintaining IL-6 levels below detectable levels at each sampling time point. When IL-6 was detected, it was at considerably lower levels than in other conditions where hepcidin is notably induced: IL-6 was typically one or more orders of magnitude higher during uncomplicated malaria [24], sepsis [45], or experimental endotoxemia [21]. Similarly, although TNF-alpha was induced, the levels detected were relatively low. Since IL-6 and TNF-alpha data were not available from the day of diagnosis or the following day, we cannot exclude the occurrence of a stronger, transient IL-6 induction during these two days. Similarly, we cannot exclude more local but significant cytokine effects in intestine, portal circulation and liver leading to hepcidin upregulation that may not be detected in systemic circulation. Nevertheless, one established feature of *S*. Typhi infection is a blunted cytokine response [40]. Several factors, most prominently the Vi-capsular polysaccharide, enable *S*. Typhi to evade innate immune responses (for example by enabling evasion of detection by complement [51]) and to establish systemic infections without clinical sepsis [40,52]. It is therefore possible signals besides IL-6 are involved in the significant acute phase response and hepcidin induction during acute typhoid infection. Thus, despite being an immunologically evasive infection, dramatic hepcidin up-regulation and hypoferremia remain features of typhoid in humans. Mechanistic links between hepcidin and hypoferremia should not, however, be concluded from observational data such as these. Nevertheless, based on data from other settings linking hypoferremia with hepcidin upregulation during infection and inflammation [11,53], we hypothesize that hepcidin plays a role in mediating hypoferremia and that the hypoferremia reflects rapid sequestration of iron in macrophages during acute typhoid infection.

The interplay between *Salmonella enterica* infection and iron has been well studied, typically through using *in vitro* or *in vivo S*. Typhimurium models. The iron exporter ferroportin is upregulated via NO-mediated Nrf2 activation in *ex vivo S*. Typhimurium-infected macrophages, reducing macrophage iron availability—a state that restricts bacterial replication [54–56]. Despite this mechanism, hepcidin induction and hypoferremia are still observed during invasive murine *S*. Typhimurium infection, associating with macrophage iron sequestration via reduced ferroportin activity; interference with hepcidin upregulation in this context, leading to reduced cellular iron levels, is protective for the host [55]. Correspondingly, hepcidin administration to infected ferroportin-expressing cells *in vitro* enhances bacterial replication [54].

As reflected by their different pathologies, there are key differences between non-typhoidal and typhoidal *Salmonella enterica* serovars, despite high degrees of sequence homology [57]. These include the expression of virulence determinants (most notably the Vi-capsular antigen) and inactivation of over 200 genes in *S*. Typhi compared with its cousin *S*. Typhimurium [57]. Interestingly, several of these inactivated genes relate to iron acquisition pathways [58]. There is evidence that *S*. Typhi relies heavily on the *fepBDCG* enterobactin ferric iron uptake system [57], which is upregulated in isolates from typhoid patients [59]; the upregulation of this system likely reflects the difficulty of obtaining iron from a host environment where iron availability is typically scarce.

In conclusion, during human *S*. Typhi infection, where hepcidin is strongly upregulated and a marked hypoferremia is observed, we hypothesize that hepcidin activity and macrophage iron retention are dominant over any macrophage cell-intrinsic protective mechanisms aimed at reducing cellular iron content. Stimulating a strong hepcidin response may represent another bacterial strategy for ensuring iron supply to facilitate replication. Therefore, in typhoid (and possibly other macrophage-tropic intracellular bacterial infections), hepcidin-induced hypoferremia may be actively disadvantageous to the host rather than being a stereotypical protective response to infection [11]. A recent study in humans demonstrated that spiegelmer-based hepcidin neutralization during experimental endotoxemia can prevent induction of hypoferremia [53]. Whether targeted manipulation of hepcidin and host iron distribution offers a potential strategy for treating intracellular infections should be investigated further, particularly in an era of increasing antibiotic resistance.

## Supporting Information

S1 Table Comparison of baseline status of study participants who became infected with typhoid with those who did not. (DOCX)

S1 Fig Baseline iron status in study participants. (A-C) Correlations of hepcidin with (A) ferritin, (B) transferrin saturation, and (C) hemoglobin concentrations in baseline, pre-typhoid challenge samples. (D-F) Comparisons of mean baseline (D) hemoglobin, (E) hepcidin, and (F) ferritin concentrations between males and females (*p*-values represent the results of t tests, based on log-transformed data for hepcidin and ferritin).
(EPS)

S2 Fig Effect of study protocol on hematological and iron parameters in individuals who challenged with *S*. Typhi, but did not develop typhoid infection. (A) Hepcidin, (B) ferritin, (C) hemoglobin concentrations, (D) red blood cell counts, (E) serum iron, (F) transferrin saturation, (G) CRP concentration and (H) temperatures were measured in the 7 individuals from Study B who were challenged with *Salmonella* Typhi but did not acquire a clinical typhoid infection. Analyte values are plotted relative to the day of typhoid challenge, day 0. (Left-hand panels) Data available from each individual for each day were plotted using box and whiskers, representing median values and interquartile ranges (IQR); whiskers represent the datapoint occurring furthest from the first or third quartile but still within 1.5*IQR of the quartile; outliers (further than 1.5*IQR from the quartile) are shown as isolated datapoints. Smoothed curves were also interpolated from the mean data for each day and overlaid on the plots. The Wald test was employed after fitting linear mixed effects models to test the null hypothesis that there is no difference between parameter values between days. Pairwise differences between baseline (day of typhoid challenge) values and other days were examined by t-tests after accounting for subject-specific variability. Significant perturbations from baseline are indicated with asterisks (*p<0.05, ***p<0.001). (Right-hand panels) Smoothed interpolated curves as described above, but depicting 95% pointwise prediction intervals (thick error bar) and conservative simultaneous Bonferroni bounds (thin error bar) of the interpolated curves.
(EPS)

S3 Fig Individual timecourses depicting changes in plasma IL-6 and TNF-alpha concentrations following *S*. Typhi challenge. Data are plotted relative to the day of typhoid diagnosis.
(EPS)

S1 Dataset Raw data. (XLSX)
