# Supplementary material for: Rapidly Escalating Hepcidin and Associated Serum Iron Starvation Are Features of the Acute Response to Typhoid Infection in Humans
Source: PLoS Negl Trop Dis. 2015 Sep 22;9(9):e0004029. doi: 10.1371/journal.pntd.0004029 (PMC4578949; doi:10.1371/journal.pntd.0004029)
Supplement: S1 Table — (DOCX) [file pntd.0004029.s001.docx]

**“S1_table.docx”: Supplementary Table S1. Comparison of baseline status of study participants who became infected with typhoid with those who did not.**

| **Parameter** | **Participants with typhoid infection (TD)** | **Participants not infected with typhoid (No TD)** |
| --- | --- | --- |
| Number of participants | 33 | 17 |
| Age, median years (IQR) | 26.1 (22.0-39.4) | 25.5 (22.3-35.2) |
| Male gender, *n* (%) | 20 (60.6) | 10 (58.8) |
| Weight, median kg (IQR) | 74.9 (70.9-80.0) | 78.15 (68.9-85.6) |
| BMI, mean (±sd) | 24.95 (±1.98) [n=27] | 25.93 (±3.38) [n=14] |
| Challenge dose, mean Log_10_ (+/- sd) | 4.29 (4.26-4.31) | 4.27 (4.26-4.29) |
| Hemoglobin, mean (+/-sd) | 14.3 (±1.5) [n=32] | 13.7 (±1.5) [n=16] |
| Mean Corpuscular Volume (MCV), | 90.5 (±4.8) [n=32] | 91.73 (±4.24) [n=16] |
| Hematocrit, | 0.432 (±0.042) [n=32] | 0.420 (±0.036) [n=16] |
| Platelets, | 243.2 (±58.1) [n=32] | 234.4 (±56.5) [n=16] |
| White cell count, | 6.3 (± 2.0) [n=32] | 6.3 (±2.1) [n=16] |
| Neutrophils, | 3.43 (±1.67) [n=32] | 3.57 (±1.79) [n=16] |
| Lymphocytes, | 2.11 (±0.70) [n=32] | 1.89 (±0.41) [n=16] |
| Eosinophils, | 0.20 (±0.12) [n=32] | 0.31 (±0.38) [n=16] |
| Serum Iron, µmol/L, | 13.4 (±4.9) | 13.3 (±6.7) |
| TIBC, µmol/L | 54.9 (±7.1) | 53.7 (±9.3) |
| Transferrin saturation (Tsat), %, | 24.9 (±10.1) | 25.2 (±11.8) |
| Log_10_ ferritin, µg/L, | 1.69 (±3.7) | 1.58 (±0.39) |
| Log_10_ hepcidin, ng/mL, | 1.04 (±0.31) | 1.06 (±0.33) |
| Log_10_ hsCRP mg/L, | -0.05 (±0.62) | -0.07 (±0.44) |

Arithmetic mean (+/- standard deviation) reported unless stated otherwise.

All comparisons returned p>0.05, t test
